# The Berlin Affective Word List for Children (kidBAWL): Exploring Processing of Affective Lexical Semantics in the Visual and Auditory Modalities

**DOI:** 10.3389/fpsyg.2016.00969

**Published:** 2016-06-30

**Authors:** Teresa Sylvester, Mario Braun, David Schmidtke, Arthur M. Jacobs

**Affiliations:** ^1^Experimental and Neurocognitive Psychology, Freie Universität BerlinBerlin, Germany; ^2^Centre for Cognitive Neuroscience, Universität SalzburgSalzburg, Austria; ^3^Center for Cognitive Neuroscience, Freie Universität BerlinBerlin, Germany

**Keywords:** kidBAWL, *Panksepp-Jakobson hypothesis*, affective semantics, negativity bias, informational density hypothesis, positivity superiority effect, valence, arousal

## Abstract

While research on affective word processing in adults witnesses increasing interest, the present paper looks at another group of participants that have been neglected so far: pupils (age range: 6–12 years). Introducing a variant of the Berlin Affective Wordlist (BAWL) especially adapted for children of that age group, the “kidBAWL,” we examined to what extent pupils process affective lexical semantics similarly to adults. In three experiments using rating and valence decision tasks in both the visual and auditory modality, it was established that children show the two ubiquitous phenomena observed in adults with emotional word material: the asymmetric U-shaped function relating valence to arousal ratings, and the inversely U-shaped function relating response times to valence decision latencies. The results for both modalities show large structural similarities between pupil and adult data (taken from previous studies) indicating that in the present age range, the affective lexicon and the dynamic interplay between language and emotion is already well-developed. Differential effects show that younger children tend to choose less extreme ratings than older children and that rating latencies decrease with age. Overall, our study should help to develop more realistic models of word recognition and reading that include affective processes and offer a methodology for exploring the roots of pleasant literary experiences and ludic reading.

## Introduction

The Berlin Affective Wordlist (BAWL; Võ et al., 2006, 2009) has been used in more than 50 studies as a means of emotion induction, diagnostics, or for investigating affective effects in perception, memory, or language. So far, all research using the BAWL and testing methodological or theoretical assumptions regarding it involved adult participants (for an overview see Jacobs et al., 2015). The present study is a first step toward providing an affective word list with highly controlled stimuli for children. Such an instrument would be of use for studying the development of human emotions and emotional intelligence (e.g., Widen and Russell, 2010; Brink et al., 2011) or the complex relationship between language and emotion in particular (Conrad, 2015; Hofmann and Kuchinke, 2015), for example in self-regulation (Vallotton and Ayoub, 2011), emotion regulation (e.g., Cole et al., 2004; Holodynski, 2013; Morawetz et al., 2016), or social understanding (Taumoepeau and Ruffman, 2008).

### Language and emotion development

The language-emotion gap, i.e., the fact that typically emotion theories are silent about language functions while linguistic theories neglect affective processes (Jacobs et al., 2015; Koelsch et al., 2015) is also apparent in developmental studies, where relatively little is known about the acquisition of an emotion lexicon and the intricate dynamic relationship between language and affect (e.g., Parladé and Iverson, 2011). Emotional development is typically studied with facial emotion recognition tasks (e.g., Mastropieri and Turkewitz, 1999). When verbal stimuli were used, often an indirect assessment procedure was applied, i.e., parents or teachers provided and rated a set of emotion words they thought to correspond to the emotion lexicon of their children/pupils (e.g., Ridgeway et al., 1985).

Such studies suggested that the domain of emotion-descriptive words has a distinctive circumplex structure organized around the two bipolar axes (valence and arousal) used by standard affective word lists for adults such as the ANEW and BAWL (Bradley and Lang, 1999; Võ et al., 2006). A direct assessment of 5–6 year old kindergartners' capacities for verbal emotion labeling using short emotion recognition vignettes (e.g., “At Christmas, Susi got a new toy that she wanted”), also indicated a good accuracy in discrete emotion (e.g., joy, fear) labeling (Ribordy et al., 1988). Both the indirect assessment of the dimensional affective structure and the direct valuation of the discrete emotion lexicon (Briesemeister et al., 2011) suggest that comparative studies are possible and may provide useful insights into the under-researched development of the affective lexicon and the dynamic interplay between language and emotion (Jacobs et al., 2015). Thus, for example, neurocognitive results for young children show that affective empathy is processed from verbal stories by the age of 8 years, but not by the age of four and that this processing differs more from that of adults compared to non-verbal affective empathy (Brink et al., 2011).

In addition, anecdotal evidence based on examples from the book “The most beautiful German word” (Limbach, 2004) and observations from daily life suggest that children are already aware of emotional and even esthetic properties of single words. Already 9-year old children can find discrete emotions, such as joy or feelings of beauty in single words and can also convincingly argue why (Schrott and Jacobs, 2011; Jacobs et al., 2015). The examples discussed in this book leave no doubt that even for small children words can be positive or negative, beautiful or ugly, more or less exciting or calming, evoke mental images of sensory-motor events, or feelings of happiness. They also support the notion of one-word poetry, i.e., that single utterances or words—even outside lyrical contexts—can fulfill what Jakobson called the “poetic function” and cause esthetic emotions (Jakobson, 1960), which have been suggested to be central to the development of pleasant literary experiences and ludic reading (Nell, 1988; Jacobs and Kinder, 2015; Jacobs, in press). Since word valence is the strongest predictor of the beauty of single words (Jacobs et al., 2015), studying valence effects with children will contribute to a better understanding of this essential literary development.

A neurocognitive account of affective word processing intended to help bridge the language-emotion gap is the so-called *Panksepp-Jakobson-Hypothesis/PJH* (Jacobs, 2015). It states that because of the relatively late appearance of language, evolution had not enough time to develop a specialized affective system for reading. Therefore, the emotional experiences during reading including esthetic feelings in literature and poetry reception, i.e., Jakobson's (1960) poetic function, are suggested to involve the activation of ancient affective circuits shared by all mammals, as best described by Panksepp (1998). For example, when words evoke the subjective feeling of disgust, the anterior insula is activated similarly to its activation in response to non-verbal stimuli (Ponz et al., 2013). Moreover, beautiful proverbs seem to activate parts of the “reward network” usually associated with food, drugs, sex, and other primary reinforcers (Bohrn et al., 2013). The *PJH* has gained multiple support from neurocognitive studies on reading and story processing (e.g., Kuchinke et al., 2005; Kissler et al., 2007; Hofmann et al., 2009; Brink et al., 2011; Altmann et al., 2012, 2014; Bohrn et al., 2012; Hofmann and Jacobs, 2014; Hsu et al., 2014, 2015a,b,c; Jacobs, 2014a,b; Briesemeister et al., 2015). It would gain further, albeit indirect, support if it was shown experimentally that children between age 6 and 12 years already show differentiated emotional effects in visual word recognition similar to those observed with adults, pointing to a close link between language and emotion early in life.

### Emotion word processing in adults: Two ubiquitous phenomena

**Figure 1:**
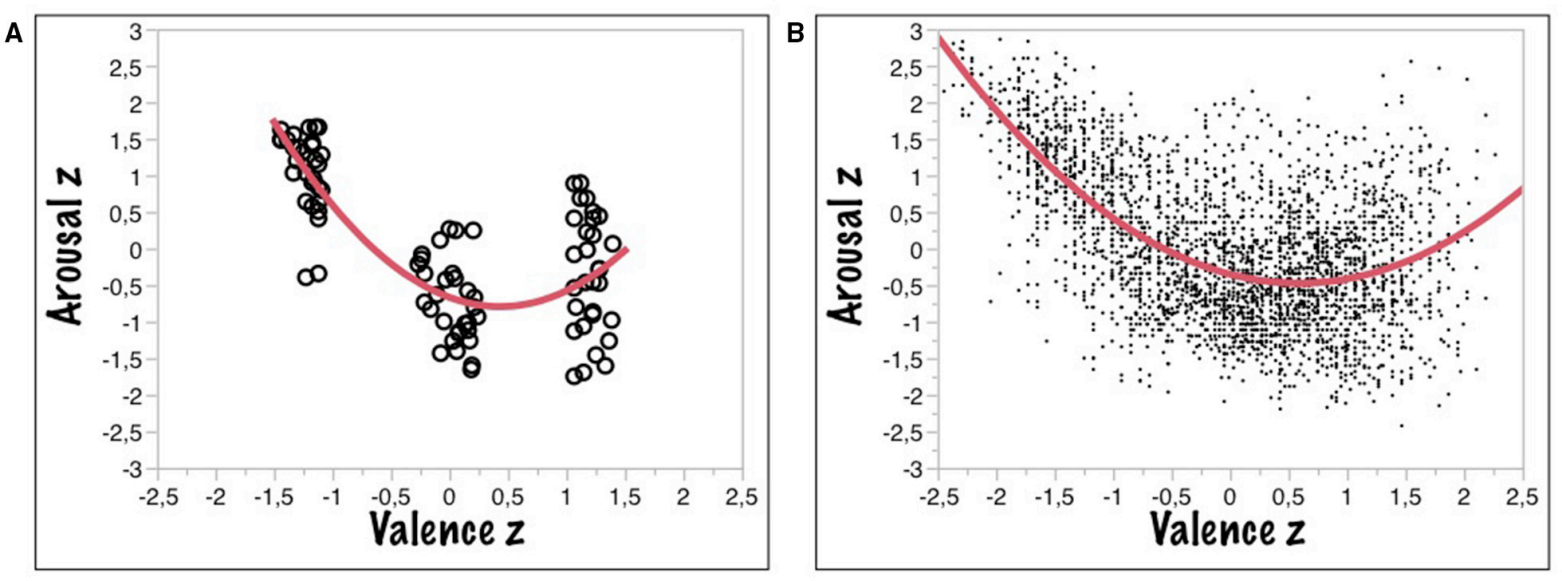
**(A)** Correlations of *z*-transformed valence and arousal values for word ratings taken from the KidBAWL. **(B)** Correlations of z-transformed valence and arousal values for word ratings taken from the BAWL (Võ et al., 2009).

The development of affective word lists such as ANEW and BAWL (Bradley and Lang, 1999; Võ et al., 2006) has helped to boost research on emotional word processing and reading in adults (for recent reviews, see Citron, 2012; Jacobs et al., 2015). Two ubiquitous phenomena have been discovered with word materials. First, the asymmetric U-shaped function relating valence ratings to arousal ratings (cf. Figure 1 below), and second, the inversely U-shaped function showing response times (RTs) in the valence decision task (VDT), which can also be slightly asymmetric (VDT; Võ et al., 2006). The first phenomenon indicates a *negativity bias*, i.e., negative words have higher arousal values than positive ones, both being more arousing than neutral ones. The second function cross-validates the explicit subjective valence ratings of words via an implicit, more objective measure (RTs) and indicates that both negative and positive words yield faster RTs than neutral ones with a slight but significant advantage for positive words (Jacobs et al., 2015). The latter can be termed the *positivity superiority effect* (Lüdtke and Jacobs, 2015).

Explanations for both these phenomena have been proposed around phylo- and ontogenetic considerations. Since aversive stimuli have arguably stronger implications for survival than do equally appetitive stimuli (e.g., avoiding a predator is more critical than pursuing a mate), the affect system may have evolved to be vigilant for and to produce rapid and strong responses to aversive and potentially harmful stimuli (Taylor, 1991; Norris et al., 2010). Both, aversive and appetitive stimuli having a higher evolutionary value than “neutral” ones, it comes as no surprise that they typically are rated of higher arousal. The “affective primacy hypothesis” states that an unattentional memory system categorizes every stimulus as positive or negative (Murphy and Zajonc, 1993), and there is evidence that this evaluation process occurs pre-consciously and incidentally at an early stage of perception (Kuchinke et al., 2005). The second ubiquitious effect, i.e., shorter RTs to positive and negative words can also be accounted for in these terms, although it does not explain why often positive words are responded to faster than negative ones, i.e., the *positivity superiority effect*. Perhaps the most general explanation of this effects is the *informational density hypothesis* which can also be applied to word processing (Ashby and Isen, 1999; Kuchinke et al., 2005; Unkelbach et al., 2008, 2010). It posits the faster processing of positive information as a function of subjective exposure frequency, that is, the experienced frequency with which positive information is internally activated in memory (i.e., processed and thought about). This subjective exposure frequency is used as a proxy for higher informational density of, for example, lexical representations of positive words, which in turn causes them to be processed faster because they are better elaborated and interconnected in memory. Recent neurocomputational evidence supports this account by showing that positive words provide more and denser semantic long-term associations than neutral or negative words (Hofmann and Jacobs, 2014). Hofmann and Kuchinke (2015) further explain the link between memory associations and (positive) valence by complementary learning systems theory (Kumaran and McClelland, 2012) and the hypothesis that the hippocampus is more generally involved in the processing of positive affect.

### The present study

While effects of dimensional and discrete affective word features are well-documented for adult subjects, we are not aware of similar studies using the ANEW, for instance, on children (cf. Jacobs et al., 2015). However, the already mentioned examples from Limbach's (2004) book and observations from daily life suggest that children are already well-aware of emotional and even esthetic properties of single words. In three experiments, we therefore investigated how 6–12 year old children process the affective semantics of words, i.e., rated written stimuli (Experiment 1), decided as fast as possible on their valence (Experiment 2), and rated spoken words (Experiment 3).

In line with the results of a pilot study reported in Jacobs et al. (2015) we expected both differences and similarities in word ratings between children and adults. On the one hand, in children of our age group, both cultural formation and lexis are less developed than in adults, and in school age brain lateralization processes are still progressing, while children learn to verbalize their or other persons' (remembered) experiences and related emotions. On the other hand, if the phylo- and ontogenetic accounts discussed above are correct, although children's affective vocabulary should be both narrower and shallower than that of adults, both ubiquitious effects reported previously should show up, at least as a tendency, also in our sample.

## Methods

### The database

The kidBAWL comprises 2045 words taken from the BAWL, particularly selected according to their suitability for use in developmental studies on language and reading acquisition and affective development in children in lower grades (age 6–12). The database includes ratings on the affective dimensions of valence and arousal as well as imageability, along with additional psycholinguistic variables used to control for in experimental contexts (Graf et al., 2005). These were number of letters (#letters), number of syllables (#syllables), number of phonemes (#phonemes), word frequency (Freq), number of orthographic neighbors (N), frequency of orthographic neighbors (FN), number of higher frequency orthographic neighbors (HFN), frequency of higher frequency orthographic neighbors (FHFN), bigram frequency (BIGmean), and syllable accent (accent). While age-of-acquisition determines one possible measure suited for approximating the age-related use of words, one major flaw constitutes mostly indirect methods of measurement such as ratings in adult cohorts (Gilhooly and Logie, 1980). We therefore attempted to further validate the suitability of our database for younger cohorts by matching entries with dictionaries particularly designed for teaching children in lower grades. Since children in this age range have a limited attention and effort span (as tested in pilot studies), we opted for a representative subsample of the kidBAWL in order to validate the database. Thus, the following experiments were restricted to 90 words randomly chosen from the kidBAWL based on the original ratings to fit with the children's attention span.

## Experiment 1. kidBAWL ratings

In a first study, 90 words were presented visually and subsequently rated by the children on the affective dimensions of valence and arousal, as well as imageability.

### Participants

In total, 20 pupils (10 female, 10 male) from seven to 12 years old (*M* = 9.2, *SD* = 1.4) participated. All pupils were native Germans without diagnosed dyslexia. The children were recruited via a primary school in Berlin.

### Material

The subset of 90 words were selected from the kidBAWL according to three valence categories of 30 positive, 30 negative, and 30 neutral affective words. Words were matched across the three valence categories on a number of features known to affect word processing: imageability (*M* = 4.25, *SD* = 1.31), letters (*M* = 6.1, *SD* = 1.26), phonemes (*M* = 5.31, *SD* = 1.12), frequency (*M* = 57.35, *SD* = 109.22), and frequency of orthographic neighbors (*M* = 1.63, *SD* = 2.25; for valence and arousal values, see Table A1 in the Appendix).

### Procedure

Words were presented in random order. For each item, the children first rated the familiarity on a 3-point scale. First appeared a statement: “The word is…,” followed by the three verbal markers “unfamiliar—partly familiar—familiar.” After entering their response, the children were presented with the statement “To me, the word feels…,” prompting them to judge the word's valence on a 5-point scale (very unpleasant—unpleasant—neither unpleasant nor pleasant—pleasant—very pleasant) as illustrated by emoticons similar to those used in the ANEW. After the response, the statement “To me, the word feels…” prompted the children to give their judgment on arousal on a simultaneously presented 5-point scale (perfectly calm—calm—neither calm nor exciting—exciting—strongly exciting) from Self-Assessment Manikins (SAM; see Jacobs et al., 2015, Supplements). Note that in order to reduce cognitive load, the scales were adapted from the original 7 and 9-point scales, respectively, to a 5-point scale for both valence and arousal. Finally, children were asked to give an imageability rating on a 3-point scale, based on three pictograms containing either nothing, a blurred or a clear stick figure.

The ratings were performed on standard laptops using PsychoPy (Peirce, 2007). Stimuli were presented in type font Times New Roman (size 40) and had a height of 1.3 cm.

The vertical visual angle was about 3.6° for the shortest and 10.2° for the longest word. If a child decided a word to be unknown, a new word was presented. The words were randomly presented to avoid primacy and recency effects. To avoid priming effects, an additional algorithm ensured that no more than three words of the same valence category were presented in series. The testing took place either in single sessions or in small groups of three children. First, an opening questionnaire was used recording age, sex, class level, and the level of tiredness. Then all children got a standardized verbal introduction to the experiment. A test run followed, where three words (banana, joy, and lecturer) were presented. Children were instructed to interrupt in case they had questions, clicked a wrong rating, or needed a break. The duration of a session varied between 18 and 42 min. Each child received a little treat as compensation.

### Results and discussion

#### Valence and arousal ratings

The ratings of all 20 children showed both significant valence and arousal effects, as established by an Linear Mixed Model (LMM) analysis with six relevant fixed effects (valence, arousal, imageability, syllables, frequency, and *N*) and two random effects (participants, words) showing that the standard (i.e., adult) valence and arousal values from the original BAWL were significant predictors of the children's valence ratings [*t*-ratio (valence) = 15.37; *p* < 0.0001; *t*-ratio (arousal) = −3.13; *p* < 0.0001], whereas only BAWL arousal was a significant predictor for the arousal ratings of the children [*t*-ratio (arousal) = 7.36; *p* < 0.0001]. Figure 1A shows that indeed the asymmetric U-shaped function relating valence ratings to arousal ratings also holds for children. Figure 1B gives the adult data from the BAWL09 study for comparison. To formally test for asymmetry, the function can be modeled with the three-free-parameter equation y = A + B^*^(x – C)^2^, where A estimates the vertical offset of the curve at its lowest point (on the y-axis), B represents the slope, and C the position on the x-axis, where the curve reaches its lowest point, i.e., an indicator of the asymmetry (0 being the theoretical minimum). This model was fit to both data sets (kidBAWL and BAWL09) yielding the results summarized in Table 1. Much as the adults' function, the children's also shows the *negativity bias*, as indicated by the positive *C*-value of 0.43 (0.58 for adults) and the obvious asymmetric shape of the theoretical (red) curve.

**Table 1:** **Three-free-parameter model fit of mean valence and arousal ratings for kidBAWL and BAWL (Võ et al., 2009)**.

	**kidBAWL**		**BAWL09**	
**General model**	f(x) = A + B^*^(x – C)^2^		f(x) = A + B^*^(x – C)^2^	
**Coefficients**	**Estimates**	**CI (95%)**	**Estimates**	**CI (95%)**
**A**	−0.81	[−1.01 – −0.70]	–0.51	[−0.55 −0.47]
**B**	0.70	[0.50 – 0.90]	0.37	[0.34 – 0.40]
**C**	0.43	[0.27 – 0.59]	0.58	[0.51 – 0.64]
**R^2^**	0.61		0.37	
**Adj**. **R^2^**	0.60		0.37	
**RMSE**	0.63		0.79	
	[*F*_(87, 2)_ = 67.4 *p* < 0.0001]		[*F*_(2899, 2)_ = 859.64 *p* < 0.0001]	

The results of correlational analyses reported elsewhere (Jacobs et al., 2015) had already established that adult valence ratings taken from the BAWL database could predict the children's ratings quite well when applied across the entire valence range (*r* = 0.91, *p* < 0.001). This suggests that in general at the level of categories (negative, neutral, positive) children of that age group have about the same concept of valence and/or the same judgment behavior as adults. Within-valence category correlations revealed a more heterogeneous picture. For the 30 negative words of the kidBAWL, only a quadratic correlation was significant (*t*-ratio = −2.1; *p* < 0.045), suggesting that children use a wider range of negative ratings including extreme values, e.g., the noun GEWALT (violence) and the verb MORDEN (to kill) had more extreme *z*-values for children than for adults (−2.2 vs. −1.4 and −2 vs. −1.4, respectively). For the 30 neutral words, the linear correlation was significant (*t*-ratio = 2.1; *p* < 0.046), whereas for the 30 positive words no significant correlation could be observed in this sample. This was due to extreme discrepancies for words like the verb KÜSSEN (to kiss) which had a much less positive *z*-value (0.3) for children than for adults (1.4). An even extremer example was the adverb OPTIMAL (optimal) with a *z*-value of 0.02 for children compared to 1.3 for adults. In contrast, the nouns MAMA (mama) or NATUR (nature) evoked more positive judgments in children (both 1.5) than in adults (both 1.2). The adult arousal ratings also predicted those of the children significantly (*r* = 0.67; *p* < 0.0001), the slightly higher values for children suggesting that either they felt more aroused by the words or were more biased toward choosing higher scale values.

#### Age, grade, and gender effects

Although not designed as a differential psychological study, we tentatively examined the potential influence of three individual factors, age, grade level, and gender on the rating data. Only gender had a significant effect on valence ratings, female pupils producing significantly higher values than males [means: 3.54 vs. 3.27; *F*_(1, 18)_ = 6.92, *p* = < 0.017; *R*^2^ = 0.28].

Due to the relatively small sample size of participants and words these results have to be interpreted with caution. They indicate that children in this age group already show the *negativity bias* in the valence - arousal function typical for adult behavior (Figure 1) and in general produce ratings suggesting a similar processing of affective lexical semantics as adults. In addition, these data raise interesting questions for future studies in this under-researched field. For example, when during development does this negativity bias first show up? Is there a general tendency for children to judge words associated with aggression or violence more negatively than adults and to rate them as more arousing? Do female pupils generally show a positivity bias as compared to male children, i.e., a tendency to use more positive valence ratings?

## Experiment 2. valence decision task with kidBAWL words

Since we had not collected any RT data in Experiment 1 and for reasons of cross-validation of the materials, we ran a second experiment with an independent sample of 47 children from Austria (Salzburg) using the VDT (with three response alternatives instead of two). It was interesting to see whether the second ubiquitious phenomenon observed in adults in a binary VDT, i.e., an inversely U-shaped function relating RTs to valence, would also appear for children, and whether children's RTs would also be shorter for both, positive and negative words, than for neutral ones with an advantage for positive words, as often observed for adults (Jacobs et al., 2015). It is of note that by using three instead of the usual two response alternatives in the VDT, we intended to make the task easier for children and reduce the hypothetical response conflict. If a clear inversely U-shaped function still showed up, this can be taken as evidence that the peak in the function is due to (semantic) word valence effects rather than to a (sensorimotor) response conflict.

### Participants

Overall 47 pupils (30 male, 17 female) between 9 and 12 years (*M* = 10.3, *SD* = 1.18) were tested. All pupils were native Austrians without diagnosed dyslexia.

The children were recruited via a daycare center in Salzburg.

### Material

The VDT used the same carefully matched 90 words (30 positive, 30 negative, and 30 neutral) as in Experiment 1. Five words of the original set had to be replaced in order to achieve a full matching of standard control variables.

### Procedure

Children decided as quickly and accurately as possible whether they judged the presented word to be of either positive, negative, or neutral valence. Stimulus presentation was randomized. A fixation cross was presented for 500 ms prior to word presentation. The word was then presented until the participant responded by a key press. The letters on the keyboard had smiley buttons: “c” (frowny), “b” (neuey), and “m” (smiley) (diameter 1.9 cm) for valence categories negative, neutral, and positive, respectively. The experiment was run on standard laptops using PsychoPy (Peirce, 2007). Words were presented in white on a black background (the letters in type font Verdana, size 40 pt).

All pupils were tested in single sessions. First they were asked to read the instruction carefully. A test run followed, where five words (sun, milk, rain, bag, and carriage) were presented. Before starting the trial, the experimenter repeated the instruction. The VDT took about 10 min on average. As in Experiment 1, the children were told that they should come forward, if they had questions, clicked a wrong button, or needed a break. Each child received a little treat as compensation.

### Results and discussion

**Figure 2:**
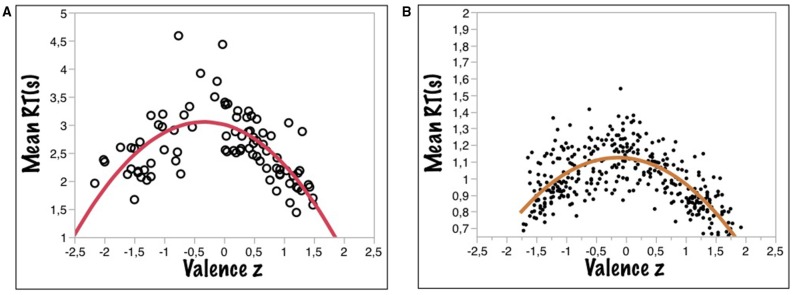
**(A)** Mean response times (RTs) as a function of mean valence ratings (z-values) for the valence decision task for data taken from the kidBAWL. **(B)** Mean response times (RTs) as a function of mean valence ratings (z-values) for the valence decision task for data taken from the BAWL (Võ et al., 2009).

Figure 2A shows that the inversely U-shaped function relating RT to valence also holds for children and the three response alternative variant of the VDT. Figure 2B gives the adult data from the BAWL06 study—using a binary VDT—for comparison. As for the rating data of Experiment 1, the function was modeled with a three-free-parameter equation [y = A – B^*^(x + C)^2^], where A estimates the vertical offset of the curve at its highest point (on the y-axis), B represents the slope, and C the position on the x-axis, where the curve reaches its lowest point, i.e., an indicator of the asymmetry (0 being the theoretical minimum). This model was fit to both data sets (kidBAWL and BAWL06) yielding the results summarized in Table 2.

**Table 2:** **Three-free-parameter model fit of z-transformed mean valence and mean reaction times (RT) for kidBAWL and BAWL (Võ et al., 2006)**.

	**kidBAWL**		**BAWL06**	
**General model**	f(x = A – B^*^(x + C)^2^		f(x) = A – B^*^(x + C)^2^	
**Coefficients**	**Estimates**	**CI (95%)**	**Estimates**	**CI (95%)**
**A**	2.99	[2.85 – 3.13]	1.12	[1.10 – 1.14]
**B**	0.40	[−0.5 – −0.30]	0.13	[−0.14 – 0.11]
**C**	0.34	[0.22 – 0.46]	0.12	[0.06 – 0.17]
**R^2^**	0.58		0.47	
**Adj**. **R^2^**	0.57		0.47	
**RMSE**	0.43		0.12	
	[*F*_(2, 86)_ = 34.18 *p* < 0.0001]		[*F*_(2, 357)_ = 161.74 *p* < 0.0001]	

**Figure 3:**
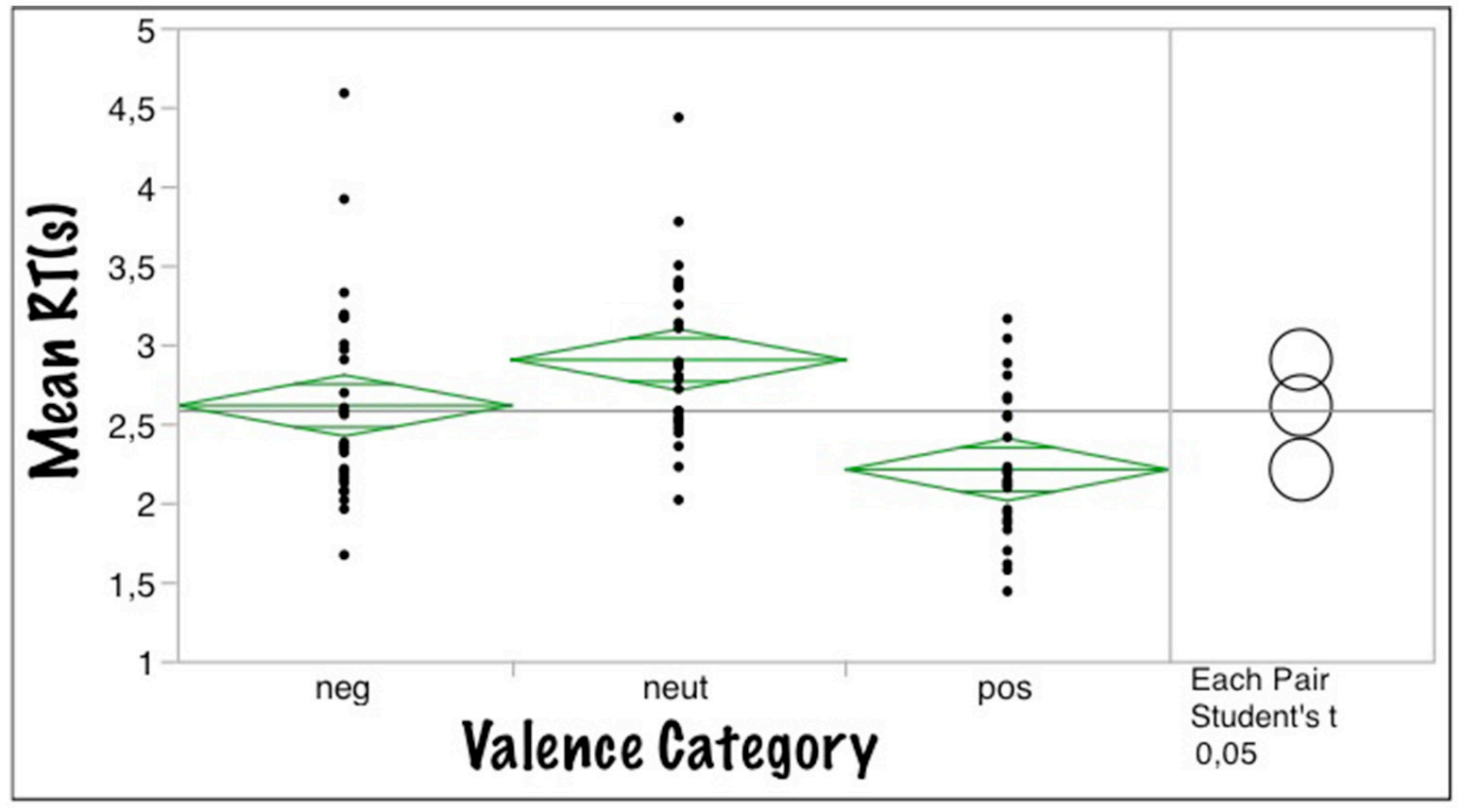
**Mean response times (RTs) as a function of valence category for the valence decision task**.

Much as the adults' function (Figure 2B), the children's (Figure 2A) also was slightly asymmetric, as indicated by the *C*-value of 0.34 (adults: 0.12). In a one-way ANOVA, valence category (negative, neutral, positive) had a significant effect on mean RTs [*F*_(2, 86)_ = 12.7; *p* < 0.0001; *R*^2^ = 0.23], *post-hoc* comparisons showing significant differences between all three categories: [Mneg = 2.61, SEneg = 0.09, Mneu = 2.9, SEneu = 0.09, Mpos = 2.20, SEpos = 0.09, pairwise differences: neg/neu: *p* = 0.034, neg/pos *p* < 0.004, neu/pos: *p* < 0.001]. Thus, pupils in that age range show the same RT rank order as adults with a clear positivity advantage, words with a positive valence being processed faster than negative ones, neutral words being slowest (see Figure 3). Notably, pupils' RTs were about 2 s slower on average than those of adults, which could be due to their relatively weaker reading skills and/or their slower processing of affective lexical semantics, or a combination of both.

The finding of longest RTs for words of neutral valence can be interpreted as showing that the peak in the inversely U-shaped function observed in previous studies on adults using the binary VDT mainly is due to semantic, i.e., word valence, effects rather than to a mere response conflict. Here, children were not forced to choose a “positive” or “negative” response for theoretically “neutral” words, but still took significantly longer to press the “neutral” response key. This suggests that the affective meaning of “neutral” words is harder to process, either because these words represent truly “neutral” stimuli (i.e., having neither clear positive nor negative connotations) and thus lacking the prioritized or higher attentional-perceptual processing of emotional stimuli (Citron, 2012), or because their connotations represent mixed emotions (i.e., having both positive and negative connotations) thus leading to a potential decision conflict resulting in prolonged responses. Further, experiments using neurocognitive methods are required to solve this theoretically interesting issue (cf. Jacobs et al., 2015), and preliminary results from a recent fMRI study point to the former alternative (Kuhlmann et al., in press).

The *positivity superiority effect* obvious in Figure 3 replicates results from previous studies on adults obtained in the VDT (Võ et al., 2006; see Figure 2B), lexical decision (e.g., Kuchinke et al., 2005), or working memory tasks (e.g., Grimm et al., 2012). This positivity advantage has been interpreted in terms of the *informational density hypothesis* outlined in the Introduction, i.e., positive stimuli being better elaborated and interconnected in memory than negative material (Ashby and Isen, 1999; Kuchinke et al., 2005; Unkelbach et al., 2008). As a tentative check of the hypothesis we ran a hierarchical cluster analysis on our kidBAWL words using the children's valence ratings (see Figure A1 in Appendix). The results indicate that positive words cluster together more strongly than negative and neutral words. If this effect can be replicated with different subjects and stimuli, it would be additional evidence that the experienced frequency with which positive information is internally activated in memory has early origins. Having shown that the *positivity superiority effect* is already present in pupils may further motivate research into its development across life span and thus provide constraints and tests of general theories of (positive) affect and emotion (e.g., Ashby and Isen, 1999).

## Experiment 3. auditory kidBAWL

Because reading can be quite stressful or tiring for children who just became literate, we also tested their processing of affective lexical semantics with auditory stimuli. Overall, we expected the results to exhibit the same two phenomena as those obtained with visual stimuli, but additionally collected RT data, this time measuring the latency of valence ratings. So far we had never measured rating latencies, but always valence decision times. We expected the rating latencies to be clearly longer than valence decision RTs, because they involve additional cognitive processes for attributing a scalar value (of valence) to each word, as well as sensorimotor ones. Moreover, the children were significantly younger and therefore would be presumably slower in overall processing.

### Participants

Thirty-two pupils (19 male, 13 female) between the age of 6 and 9 years (*M* = 7.77; *SD* = 0.91) were tested. The children were recruited from a mixed age class in a primary school in Berlin. All pupils' parents signed a letter of agreement.

### Material

The 90 carefully matched words of Experiment 1 were re-used here plus 15 randomly chosen, matched words from the kidBAWL, five of each valence category (positive, negative, and neutral). The stimulus set was divided into one base set of 60 words and an additional set of 40 words to ensure that each child rated the base set in case of early interruptions.

### Procedure

The pupils were tested during class in a separate room in single sessions. First, an opening questionnaire was used. Pupils were asked for their age, gender, grade level, and tiredness. Within a test run, five words (threat, strawberry, fear, curiosity, and fun) were presented, testing for comprehension of the SAMS's by standardized instructions. Starting testing, words were presented in random order within the sets. Words were presented auditorily by circumaural earphones, the volume being individually adapted to pupils' preference. Simultaneously, upon auditory stimulus presentation, a simple binary scale appeared where pupils answered the question “Do you know the word?” choosing either “yes” or “no.” Before answering, participants could repeat each word if needed by clicking a speaker symbol just above. In case a word was unknown, the trial was skipped, otherwise, a 5-point valence scale appeared below, implemented via pictorial SAMs similar to Experiment 1, to prompt the pupils to rate the valence by using a mouse. Response latencies were measured starting from scale presentation. After pressing a button, the SAM's 5-point arousal scale appeared prompting the children to give an arousal judgment. All sessions were performed on standard laptops (i.e., 13.3 inch Vaio, Windows 7, 1366 × 768 pixel, 2.13 GHZ), using a Java based program. After the first set of 60 words a short break was taken. The sessions lasted between 16 and 48 min (*M* = 28, *SD* 7.44). The pupils received strawberries as compensation.

### Results and discussion

Two pupils were excluded from analysis, one because of extremely low concentration and inappropriate behavior, the other because of a biased response tendency to use extreme ratings only. Furthermore, five words were excluded because they were known by less than 51% of the participants: bankrott (bankrupt: 30%), Diktatur (dictatorship: 43%), Justiz (justice: 23%), optimal (optimal: 50%), Tumor (tumor: 27%).

#### Valence and arousal ratings

**Figure 4:**
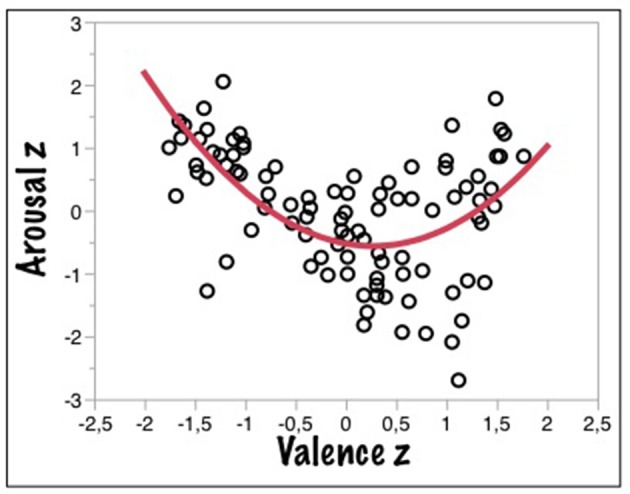
**Correlations of z-transformed mean valence and arousal values for the auditory kidBAWL**.

Figure 4 shows that the asymmetric U-shaped function relating valence to arousal ratings also holds for auditory stimuli, thus providing a novel cross-modal cross-validation for the visual data from Experiments 1 and 2. Valence ratings in both modalities (compared with the ratings from Experiment 1) were highly correlated [*r* = 0.94, *F*_(83, 1)_ = 659.52, *p* < 0.0001]. The correlation for arousal was smaller, but also significant [*r* = 0.71, *F*_(81, 1)_ = 85.15, *p* < 0.0001]. The results for the three free parameter model are summarized in Table 3.

**Table 3:** **Three-free-parameter model fit of mean valence and arousal ratings for auditory kidBAWL**.

**Valence × arousal kidBAWL**		
**General model**	f(x) = A + B^*^(x – C)^2^	
**Coefficients**	**Estimates**	**CI (95%)**
**A**	−0.53	[−0.77 – 0.28]
**B**	0.55	[0.36 – 0.74]
**C**	0.25	[0.07 – 0.43]
**R2**	0.34	
**Adj**. **R^2^**	0.33	
**RMSE**	0.82	
	[*F*_(2, 93)_ = 23.87 *p* < 0.0001]	

#### Valence rating latency (RTs)

**Figure 5:**
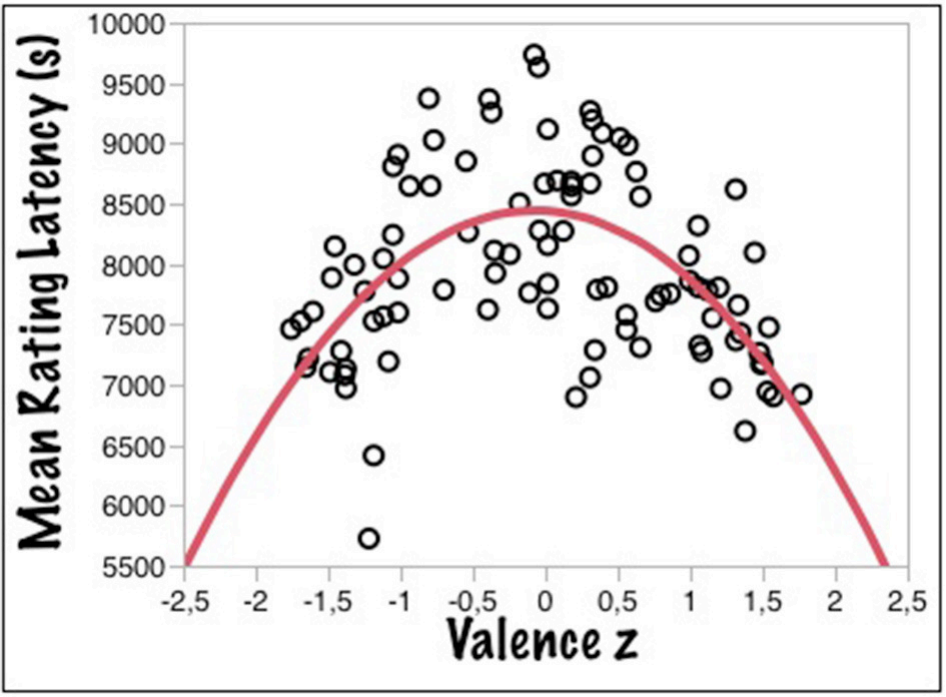
**Mean rating latencies (RTs) as a function of z-transformed mean valence ratings for the auditory kidBAWL**.

A second novelty of Experiment 3 was the measurement of valence rating latencies. The data are summarized in Figure 5, showing that the typical inversely U-shaped function found in the binary VDT (Experiment 2) also holds for rating latencies^1^ with the notable difference that parameter A is much higher. This indicates that latencies are about 2.5 times slower than binary decisions, the extra time being due to processes attributing a rating value to each word, extra sensorimotor time, and possibly also to the significantly younger age of the children compared to those of the previous studies. The results for the three-free-parameter model are summarized in Table 4.

**Table 4:** **Three-free-parameter model fit of ***z***-transformed mean valence × mean rating latencies (RT) for auditory kidBAWL**.

	**Mean rating latency × valence kidBAWL**	
**General model**	f(x) = A – B^*^(x + C)^2^	
**Coefficients**	**Estimates**	**CI (95%)**
**A**	8443	[8244 – 8642]
**B**	490.1	[341.1 – 639
**C**	0	
***R***^**2**^	0.34	
**Adj**. ***R***^**2**^	0.33	
**RMSE**	659.6	
	[*F*(2, 93) = 23.67 *p* < 0.0001]	

Overall, the results summarized in Figures 4, 5 cross-validate those of Experiments 1 and 2 indicating that for the present age group and stimuli, valence, and arousal ratings are virtually independent of presentation mode, while valence rating latencies in the auditory modality are slowed by a factor of about 2.5 with regard to RTs in the VDT.

#### Age and gender effects

There was a significant effect of age on mean valence rating latencies [*RT* = 14546 – 832 × age; *F*_(1, 27)_ = 5.67, *p* < 0.024] reflected by the following rank order: 6 years: 9935 ms, 7 years: 8573 ms, 8 years: 7681 ms, and 9 years: 7321 ms, but no effect of gender.

## General discussion

The BAWL (Võ et al., 2006, 2009) represents one of the most widely used affective dictionaries for the German language area (Jacobs et al., 2015). To enable research on developmental questions concerning human emotions and emotional intelligence (e.g., Brink et al., 2011) or the complex relationship between emotion and language (e.g., Conrad, 2015) we developed an adaption for research with children in lower grades (age 6–12), providing ratings on the affective dimensions of valence and arousal, as well as imageability along with additional psycholinguistic variables. In a first attempt to investigate the processing of affective meaning in words by 6–12 year olds, we conducted three experiments in which children rated visually (Experiment 1) and auditorily (Experiment 3) presented words on valence and arousal, and additionally decided on valence in a VDT (Experiment 2) in order to further cross-validate subjective ratings by means of the more objective RT measure.

In general, two phenomena most prominent in research on affective word processing and reading in adults could be replicated in our studies with children. First, concerning the relation between valence and arousal, an asymmetric U-shaped distribution across the two dimensions including a characteristic *negativity bias* was replicated in both visual and auditory domains, as had already been observed in numerous studies on adults (e.g., Võ et al., 2009; Schmidtke et al., 2014). Thus, stronger correlations within the ranges of either positive or particularly negative valence add further evidence against the assumption of valence and arousal as two fully independent components of a two-dimensional affective space, despite weaker correlations across the whole range of the valence spectrum. Second, with neutral words being processed more slowly than negative or positive ones, also a characteristic inversely U-shaped function relating RTs to valence was observed in a VDT as well as in ratings in the auditory domain. We also replicated another asymmetry in terms of a slight processing advantage for positive compared to negative words as had already been observed in adults: the *positivity superiority effect* (Võ et al., 2006; Mueller and Kuchinke, 2016).

As was expected, there were also large differences in response latencies, children's RTs being significantly slower than those of adults. This is likely due to their lesser reading skills yet to be developed, as suggested by a significant decrease of response latencies with increasing age (Experiment 3). However, our data strongly suggest that even at the onset of reading acquisition, children are already well-aware of the affective connotations contained in linguistic material such as single words. Of theoretical importance here is the issue how words acquire affective meaning in the first place (e.g., Braun, 2015). Since language and emotion appear to be linked via phylogenetically old brain systems (Panksepp, 2008a; Jacobs, 2015), an evolutionary explanation in terms of the PJH discussed above seems plausible. Regarding ontogenetic development, phylogenetically bound subcortical affect systems in interaction with epigenetic factors and learning processes thus may guide and constrain the development of neocortical functional networks that according to Panksepp (2008b) may otherwise resemble a tabula rasa at birth. However, as Koelsch et al. (2015) suggest, it is not just communicative skills in terms of both expression and comprehension but also the regulation of emotions that represents an important element of the language-emotion nexus. In this context, it is most prominently neocortical functions developed in phylogenetically as well as ontogenetically later stages, that feed back to older basic affect systems and accordingly build highly integrated networks that constitute the basis of complex cognitive-emotional behaviors such as language. The simple fact that the children's vocabulary can be expected to be narrower and shallower than that of adults may therefore go along with not yet acquired or less refined social concepts and still ongoing internalization of complex social norms as these themselves may be bound to linguistic competence (Rose-Krasnor, 1997). This could explain, for example, higher rating values in negative high arousing concepts or a larger amount of variance in the rating values in general as compared to adult ratings.

Clearly, such speculations call for more efforts toward closing the language-emotion gap with a special focus on experimental developmental studies. The present kidBAWL database provides a novel means for future comparative studies that may offer useful insights into so far neglected research issues concerning the development of the affective lexicon, i.e., how words acquire affective meaning in the first place, and the highly complex interplay between language and emotion. This should be helpful for informing and constraining theoretical models of word recognition and reading acquisition (e.g., Grainger and Jacobs, 1996; Perry et al., 2007; Hofmann and Jacobs, 2014) that so far neglect affective and esthetic processes altogether (Jacobs and Kinder, 2015; Jacobs et al., 2015), as well as theories of affect (e.g., Ashby and Isen, 1999).

## Ethics statement

The parents of the children signed a written consent. Additionally the children were asked if they would like to participate. Theywere told that they can interupt the experiement whenever they like to, without any consequences.

## Author contributions

TS: Experiment 1. AJ: Experiment 1. MB: Experiment 2. DS: Experiment 3.

### Conflict of interest statement

The authors declare that the research was conducted in the absence of any commercial or financial relationships that could be construed as a potential conflict of interest.

## Footnotes

^1^It has been reported before that the average time to *classify* a stimulus as positive or negative correlates highly with the time required to *rate* a stimulus, e.g., on a 5-point scale (Unkelbach et al., 2008).
