# Supplementary material for: The Berlin Affective Word List for Children (kidBAWL): Exploring Processing of Affective Lexical Semantics in the Visual and Auditory Modalities
Source: Front Psychol. 2016 Jun 30;7:969. doi: 10.3389/fpsyg.2016.00969 (PMC4928334; doi:10.3389/fpsyg.2016.00969)

**Appendix**

**Table A1**. Word stimuli with valence and arousal values.

| **Word** | **Mean Valence** | **Mean Arousal** |
| --- | --- | --- |
| AHNEN | -.02 | -.36 |
| ANGST | -.54 | .07 |
| ARZT | .5 | .25 |
| AUSREDEN | .24 | -.24 |
| BANKROTT | -.57 | -.43 |
| BASIS | .11 | -.38 |
| BATTERIE | .1 | -.1 |
| BEGRABEN | -.44 | -.24 |
| BELIEBT | .42 | .18 |
| BESTIE | -.75 | .54 |
| BETRUG | -1.25 | .4 |
| BETT | .62 | -.88 |
| BLUME | .83 | -.6 |
| CHARME | .08 | -.69 |
| DIKTATUR | -.31 | 0 |
| DROHEN | -1.04 | .39 |
| EINTRETEN | -.05 | -.09 |
| ELEND | -1.02 | .07 |
| ENGEL | .69 | -.41 |
| FASSEN | .12 | -.09 |
| FEIND | -.89 | .29 |
| FERIEN | .85 | .42 |
| FLEISCH | .27 | -.41 |
| FLUCH | -.74 | .29 |
| FREIZEIT | 0.83 | -.29 |
| FREUEN | .81 | -.19 |
| FREUND | .87 | -.1 |
| FRÜHJAHR | .72 | -.24 |
| FÜHLEN | .46 | .07 |
| GEISEL | -1.08 | .67 |
| GENIAL | .58 | .07 |
| GESCHENK | .96 | .33 |
| GEWINN | .62 | .64 |
| GIFT | -1.08 | .37 |
| GRAUSAM | -.89 | .29 |
| GUMMI | .3 | -.33 |
| HARMONIE | .51 | -.55 |
| HASSEN | -.97 | .07 |
| HERZ | .73 | .07 |
| INSEKT | .16 | -.05 |
| PARTY | .77 | .77 |
| PUMPE | .08 | -.45 |
| JUSTIZ | -.12 | -.15 |
| KILLER | -.6 | .83 |
| KIRSCHE | .58 | -.24 |
| KÜSSEN | .15 | .2 |
| LÄCHELN | .95 | -.6 |
| LEICHE | -1.43 | .72 |
| LEITER | .35 | -.41 |
| LIFT | .11 | -.41 |
| LOB | .73 | -.04 |
| MAMA | 1 | -.1 |
| METER | .31 | -.23 |
| MIETEN | -.01 | -.24 |
| MORDEN | -1.44 | .67 |
| MUTIG | .58 | .59 |
| NATUR | 1 | .07 |
| NOTE | .46 | .64 |
| OPTIMAL | -.02 | -.31 |
| RACHE | -1.01 | .25 |
| REVIER | 0.17 | -.04 |
| RUDER | .26 | -.24 |
| SARG | -.41 | .07 |
| SCHEUSAL | -.8 | -.3 |
| SCHLAFEN | .61 | -.97 |
| SCHWUR | .25 | .17 |
| SELTSAM | -.03 | .04 |
| SPRUNG | .42 | -.15 |
| SKLAVE | -.94 | .29 |
| STEHLEN | -1.16 | .27 |
| TERROR | -.5 | .58 |
| TOPFIT | .88 | -.21 |
| TOLL | .81 | -.19 |
| TRAGISCH | -.62 | -.03 |
| TRAURIG | -1.21 | -.36 |
| TRIUMPH | .28 | -.15 |
| TUMOR | -.89 | .29 |
| UNFALL | -1.04 | .36 |
| UNHEIL | -.76 | -.01 |
| URTEIL | -.15 | -.21 |
| VERSAGER | -1.12 | .03 |
| WERBUNG | -.58 | -.45 |
| WINTER | .39 | -.1 |
| ZÄHLEN | .35 | -.1 |
| ZUKUNFT | .31 | .33 |
| ZWIEBACK | .35 | -.54 |

**Figure A1**. Constellation plot of a hierarchical cluster analysis (Ward method) using mean valence as input variable (positive words in green, negative in blue, neutral in red).


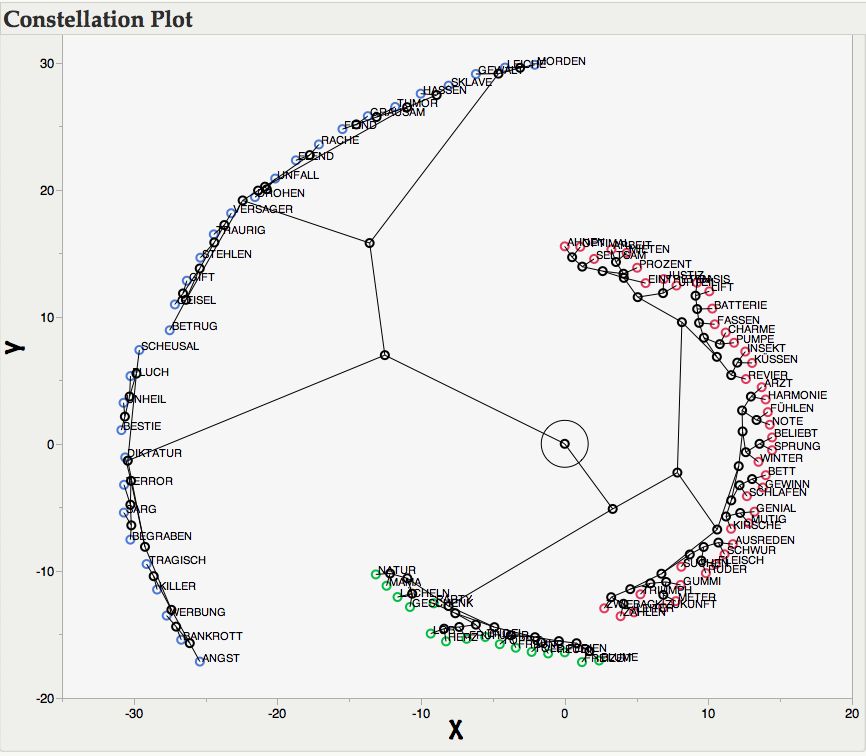

Supplement: Supplementary file 1 [file DataSheet1.docx]
